# Identifying women with gestational diabetes based on maternal characteristics: an analysis of four Norwegian prospective studies

**DOI:** 10.1186/s12884-021-04086-9

**Published:** 2021-09-08

**Authors:** Anam Shakil Rai, Line Sletner, Anne Karen Jenum, Nina Cecilie Øverby, Signe Nilssen Stafne, Tove Lekva, Are Hugo Pripp, Linda Reme Sagedal

**Affiliations:** 1 Department of Research, Sorlandet Hospital, 4604 Kristiansand, Norway; 2 Department of Pediatric and Adolescents Medicine, Akershus University Hospital, Akershus, Norway; 3 Institute of Clinical Medicine, University of Oslo, Oslo, Norway; 4 Department of General Medicine, General Practice Research Unit (AFE), Institute of Health and Society, University of Oslo, Oslo, Norway; 5 Department of Nutrition and Public Health, Faculty of Health and Sport Sciences, University of Agder, Kristiansand, Norway; 6 Department of Public Health and Nursing, Norwegian University of Science and Technology (NTNU), Trondheim, Norway; 7 Department of Clinical Services, St.Olavs Hospital Trondheim University Hospital, Trondheim, Norway; 8 Research Institute of Internal Medicine, Oslo University Hospital, Oslo, Norway; 9 Oslo Centre of Biostatistics and Epidemiology, Research Support Services, Oslo University Hospital, Oslo, Norway; 10 Department of Obstetrics and Gynaecology, Sorlandet Hospital, Kristiansand, Norway

**Keywords:** Gestational diabetes mellitus, Pre pregnancy BMI, Pregnancy, Screening, Diagnostic criteria

## Abstract

**Background:**

There is still no worldwide agreement on the best diagnostic thresholds to define gestational diabetes (GDM) or the optimal approach for identifying women with GDM. Should all pregnant women perform an oral glucose tolerance test (OGTT) or can easily available maternal characteristics, such as age, BMI and ethnicity, indicate which women to test? The aim of this study was to assess the prevalence of GDM by three diagnostic criteria and the predictive accuracy of commonly used risk factors.

**Methods:**

We merged data from four Norwegian cohorts (2002–2013), encompassing 2981 women with complete results from a universally offered OGTT. Prevalences were estimated based on the following diagnostic criteria: _1999_WHO (fasting plasma glucose (FPG) ≥7.0 or 2-h glucose ≥7.8 mmol/L), _2013_WHO (FPG ≥5.1 or 2-h glucose ≥8.5 mmol/L), and _2017_Norwegian (FPG ≥5.3 or 2-h glucose ≥9 mmol/L). Multiple logistic regression models examined associations between GDM and maternal factors. We applied the _2013_WHO and _2017_Norwegian criteria to evaluate the performance of different thresholds of age and BMI.

**Results:**

The prevalence of GDM was 10.7, 16.9 and 10.3%, applying the _1999_WHO, _2013_WHO, and the _2017_Norwegian criteria, respectively, but was higher for women with non-European background when compared to European women (14.5 vs 10.2%, 37.7 vs 13.8% and 27.0 vs 7.8%). While advancing age and elevated BMI increased the risk of GDM, no risk factors, isolated or in combination, could identify more than 80% of women with GDM by the latter two diagnostic criteria, unless at least 70–80% of women were offered an OGTT. Using the _2017_Norwegian criteria, the combination “age≥25 years or BMI≥25 kg/m^2^” achieved the highest sensitivity (96.5%) with an OGTT required for 93% of European women. The predictive accuracy of risk factors for identifying GDM was even lower for non-European women.

**Conclusions:**

The prevalence of GDM was similar using the _1999_WHO and _2017_Norwegian criteria, but substantially higher with the _2013_WHO criteria, in particular for ethnic non-European women. Using clinical risk factors such as age and BMI is a poor pre-diagnostic screening method, as this approach failed to identify a substantial proportion of women with GDM unless at least 70–80% were tested.

**Supplementary Information:**

The online version contains supplementary material available at 10.1186/s12884-021-04086-9.

## Background

Gestational diabetes mellitus (GDM) is glucose intolerance with onset or first diagnosis during pregnancy which is clearly not overt diabetes [1]. GDM is associated with higher maternal and neonatal morbidities in the short- and long-term and predisposes both women and their offspring to later development of type 2 diabetes [2]. Screening followed by treatment of GDM reduces the risk of several pregnancy complications [3]. However, there is no worldwide agreement on the best diagnostic thresholds to define GDM, and a wide variety of clinical guidelines have been employed [4].

In 2013, the World Health Organization (WHO) recommended glycaemic thresholds for the diagnosis of GDM based on findings from the multinational Hyperglycaemia and Adverse Pregnancy Outcome (HAPO) study demonstrating a linear dose-response between maternal glycaemia and adverse neonatal outcomes. These criteria were determined to identify women with an adjusted odds ratio (OR) of 1.75 for adverse events in their offspring relative to the mean [5]. Glucose values set to identify women with a higher risk, corresponding to an adjusted OR of 2.0, were also considered but this proposal was rejected. Nonetheless, several countries, among them Canada and Norway, adopted the latter noting the substantial rise in GDM prevalence by _2013_WHO criteria, without clear evidence of clinically important benefits [6]. The prior WHO criteria, established in 1999 and used in Norway until 2017, were identical to those for diagnosis of glucose intolerance in a non-pregnant population.

Controversy surrounds not only the thresholds values of glycemia, but also the optimal approach for identifying women with GDM. A high-risk approach has traditionally been recommended based on easily available maternal characteristics such as advanced age and BMI, known to be associated with an increased risk of GDM [7]. However, although this approach reduces unnecessary testing in those least likely to test positive, a key issue is their performance as indicators for diagnostic testing and the usefulness of risk factors in a clinical setting today [8]. The alternative option, universal screening, has a high detection rate but poses a large immediate burden to healthcare services as well as pregnant women.

In this study that merged data from four existing Norwegian pregnancy and birth cohorts, we aimed to address some of the clinical controversies related to GDM diagnosis and screening. The objectives were: 1) To establish the prevalence of GDM with three diagnostic criteria (_1999_WHO, _2013_WHO, and the _2017_Norwegian criteria), 2) identify cut-off levels for age and BMI that identify at least 80% of women with GDM and 3) assess the predictive accuracy of commonly used risk factors.

## Methods

All population-based birth cohort studies in Norway with a special focus on gestational diabetes were eligible. For the present study, the following inclusion criteria were defined: (i) prospective studies comprising women with singleton live-born children recruited early in pregnancy (between week 15–20); (ii) data on maternal pre-pregnancy BMI; (iii) glucose measurements obtained from at least one universally offered 75 g 2-h oral glucose tolerance test (OGTT) performed ≥20 weeks’ gestation; (iv) at least one offspring measurement (birthweight). Exclusion criteria were studies without the core data and studies that only included specific subgroups (such as obese women only).

**Fig. 1:**
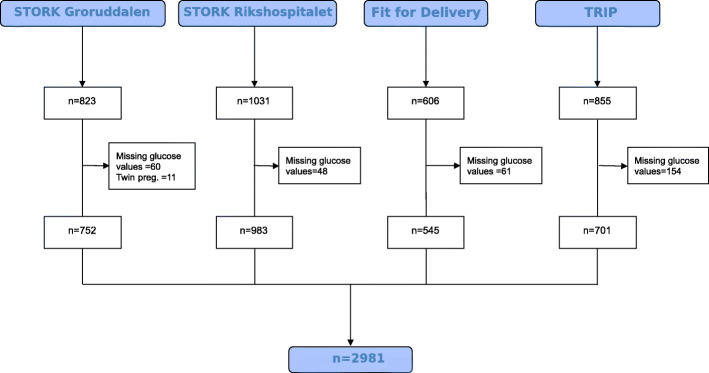
Flowchart of included studies and excluded participants from each study

Four Norwegian studies (two cohort studies ( [9, 10] and two randomized controlled trials (RCT) [11, 12] were identified, and primary investigators were invited to become part of the “Norwegian Hyperglycemia in pregnancy” consortium in 2017. Principal investigators from all four studies agreed to participate, providing data from 3315 pregnant women and 3293 live births (Fig. 1).

The original studies collected data between 2002 and 2013. If GDM was diagnosed, women received diabetes care according to local guidelines. Details of the methods and characteristics of participants in each study, including eligibility criteria, methods of recruitment and measurements obtained, have been previously published [9–12]. Authors were requested to provide anonymous raw data to be stored and analyzed in The University of Oslo’s Service for Sensitive data (TSD) storage platform with access for all the project partners. Data were further harmonized and assessed for internal consistency and missing items. Investigators were asked for clarification on issues regarding the coding of variables and a final summary of relevant variables was sent for verification. After resolution, all datasets were merged. We excluded from analyses participants for which no OGTT data were available, as well as multi-fetal pregnancies (Fig. 1).

The primary outcome was GDM prevalence. All women underwent a 75 g OGTT after an overnight fast. In two of the studies [9, 10] venous blood samples were collected in tubes containing Ethylenediaminetetraacetic acid (EDTA) and glucose was analyzed on site in fresh, whole EDTA blood, using HemoCue 201+ glucose analyser (Angelholm, Sweden) [9] or a Accu-Chek Sensor glucometer (Roche Diagnostics, Mannheim, Germany) [10] according to protocols. In Sagedal et al. [11] and Stafne et al. [12] fasting and 2-h glucose levels were measured in plasma or serum, respectively, by the routine methods used at the participating hospital laboratory.

The diagnosis was originally made according to the _1999_WHO criteria which was used during data collection. In addition, we applied the _2013_WHO criteria and the _2017_Norwegian criteria (Table 1) for the purposes of this specific study. The _2013_WHO criteria also includes a 1-h glucose which was not measured in the respective studies.

**Table 1:** Criteria for gestational diabetes

Glucose value	_1999_WHO	_2013_WHO	_2017_Norwegian
Fasting	≥7.0 mmol/L	≥5.1 mmol/L	≥5.3 mmol/L
2-h	≥ 7.8 mmol/L	≥8.5 mmol/L	≥9.0 mmol/L

Based on a 75 g Oral Glucose Load. For the diagnosis, one or more of the glucose values must be met or exceeded

In each individual study, women were either interviewed or asked to complete a questionnaire including information on current smoking status and their highest educational qualification. Women were further assessed at the study sites with respect to biological and anthropometric data. Height was measured directly while weight prior to becoming pregnant was self-reported in all studies. Categories for age and pre-pregnancy body mass index (BMI) were determined prior to analysis and based on clinical relevance. Furthermore, women were classified as primiparous or parous for the purpose of this study.

STORK Groruddalen [9] was the only study that actively included a multiethnic population (59% ethnic minority women, primarily born outside Europe). Ethnic origin was defined as European (predominantly Scandinavian as well as East and West-European origin) or non-European (mainly Asian, North-African, Middle Eastern or Sub-Sahara African). Family history of diabetes was not measured in the Fit for Delivery study.

### Statistical analysis

Distributions of all potential predictors were checked for normality. The characteristics of the women were categorized by GDM-status and the two groups were compared using X^2^ statistic for categorical data and the Student’s t Test for continuous variables. Data are reported as frequencies and percentages for categorical variables and mean and standard deviation for continuous variables.

Information was available for 95% of the selected covariates. To assign values for the missing data for pre-pregnancy weight (5%), height (0.4%), educational attainment (0.3%) and parity (0.3%) we used Stochastic regression imputation with predictive mean matching as the imputation model to substitute missing items in the observed population [13].

To examine associations between GDM and maternal factors, we modelled GDM as a binary outcome (GDM vs no-GDM) and variables related to GDM in univariable logistic regression models with *p*-value < 0.2 were considered in separate multivariable analyses [14]. The final model resulted from a backward selection procedure (exclusion if *p* > 0.15)*.* All models were adjusted for cohort to handle unmeasured confounders. Results from logistic regression are presented as OR with accompanied 95% confidence intervals (CI), and with Nagelkerke R^2^ for model fit.

In the analyses, the two RCT’s were treated as cohort studies as the primary outcome (GDM) did not differ between control and intervention group in the original studies [11, 12]. The interventions in the two trials consisted of either an exercise program (supervised exercise sessions) or a combination of a physical activity component and dietary counselling. The regression analysis was repeated after excluding participants who received the intervention to examine the potential role of the intervention in these RCTs.

Finally, we assessed the diagnostic accuracy across different pre-specified cut-offs for maternal age and BMI with and without the addition of parity, based on previous and current screening guidelines. We calculated sensitivity (proportion of GDM cases correctly identified by the risk factor), specificity (proportion of women without GDM who did not have the risk factor), and the proportion of women with the risk factor (i.e. who would be offered an OGTT). Analyses were performed and presented separately for European and non-European women due to strong effect of ethnicity. For each risk factor, single or in combination, the sensitivity estimates were plotted in Receiver Operating Characteristic (ROC) space against the proportion of women subjected to OGTT. An optimal risk factor combination will have high sensitivity with small numbers needing to be tested (results near the top left of the space). We opted for a sensitivity level of 80% for the risk factors. Statistical analyses were performed using SPSS software, Version 26 (USA).

## Results

We excluded more participants from the TRIP study than from the other studies due to missing GDM data (Fig. 1). Apart from this, no significant differences were noted between the women who were included in the study and those excluded (not shown). After exclusions, the pooled dataset comprised 2981 women with a mean (SD) age of 30.2 (4.4) years and pre-pregnant BMI of 23.7 kg/m^2^ (Table 2). The majority were of European origin (87.0%), had higher education (73.4%) and were in their first pregnancy (61.0%). GDM was diagnosed in 320 (10.7%), 504 (16.9%) and 308 (10.3%) pregnancies with the _1999_WHO, _2013_WHO and _2017_Norwegian criteria, respectively.

**Table 2:** Characteristics of the participating pregnancy and birth cohorts

Characteristics	Stork Grorudddalen	Stork Rikshospitalet	Fit for Delivery	TRIP	Total
	*n* = 752	*n* = 983	*n* = 545	*n* = 701	*n* = 2981
**Study period**	2008–2010	2002–2008	2009–2013	2007–2009	
**Type of study**	cohort	cohort	RCT	RCT	
**Gest. age at inclusion (weeks)**	15.1 ± 3.4	15.8 ± 1.3	15.1 ± 2.6	20.2 ± 1.6	16.5 ± 3.1
**Gest. age at OGTT (weeks)**	28.3 ± 1.3	31.2 ± 1.0	29.6 ± 0.8	34.0 ± 2.0	30.8 ± 2.5
**European ethnicity**	363 (48.3)	983 (100)	541 (99.3)	701 (100)	2588 (86.8)
**Current smoker**	31 (5.0)	23 (2.3)	20 (3.7)	6 (0.9)	80 (2.8)
**Education**					
**primary or less**	124 (16.5)	12 (1.2)	10 (1.8)	3 (0.4)	149 (5.0)
**High school education**	297 (39.5)	128 (13.0)	158 (29.0)	62 (8.8)	645 (21.6)
**Higher education**	331 (44.0)	843 (85.8)	377 (69.2)	636 (90.7)	2187 (73.4)
**Primipara**	345 (45.9)	524 (53.3)	545 (100)	405 (57.8)	1819 (61.0)
**Diabetes in family**	191 (26.1)	98 (10.5)	NM	61 (9.1)	350 (11.7)
**Age (years)**					
Total	29.9 ± 4.8	31.3 ± 3.8	28.0 ± 4.3	30.6 ± 4.2	30.2 ± 4.4
Primipara	28.1 ± 4.6	30.0 ± 3.7	28.0 ± 4.3	29.2 ± 3.7	28.9 ± 4.1
Parous	31.4 ± 4.5	32.7 ± 3.6	^a^	32.4 ± 4.1	32.2 ± 4.0
**Prepregnant BMI (kg/m**^**2**^**)**	24.6 ± 4.8	23.4 ± 3.7	23.6 ± 3.8	23.1 ± 3.1	23.7 ± 3.9
**BMI at inclusion (kg/m**^**2**^**)**	25.3 ± 4.8	24.5 ± 3.4	24.5 ± 3.9	24.7 ± 3.1	24.8 ± 4.0
**Fasting glucose at OGTT (mmol/L)**	4.8 ± 0.6	4.6 ± 0.4	4.6 ± 0.4	4.3 ± 0.4	4.6 ± 0.5
**2-h glucose at OGTT (mmol/L)**	6.2 ± 1.4	6.2 ± 1.4	6.1 ± 1.3	5.7 ± 1.2	6.1 ± 1.3
**GDM,**_**1999**_**WHO-criteria**	97 (12.9)	124 (12.6)	57 (10.5)	42 (6.0)	320 (10.7)
**GDM,**_**2013**_**WHO-criteria**	236 (31.4)	145 (14.8)	76 (13.9)	47 (6.7)	504 (16.9)
**GDM,**_**2017**_**Norway-criteria**	156 (20.7)	87 (8.9)	38 (7.0)	27 (3.9)	308 (10.3)

Data presented as mean ± SD or n (%). Values are imputed for pre-pregnancy weight, parity and education

^a^*Only primipara included in the study*

*OGTT* oral glucose tolerance test, *BMI* body mass index, *GDM* gestational diabetes mellitus, *WHO* World Health Organization, *NM* not measured

**Fig. 2:**
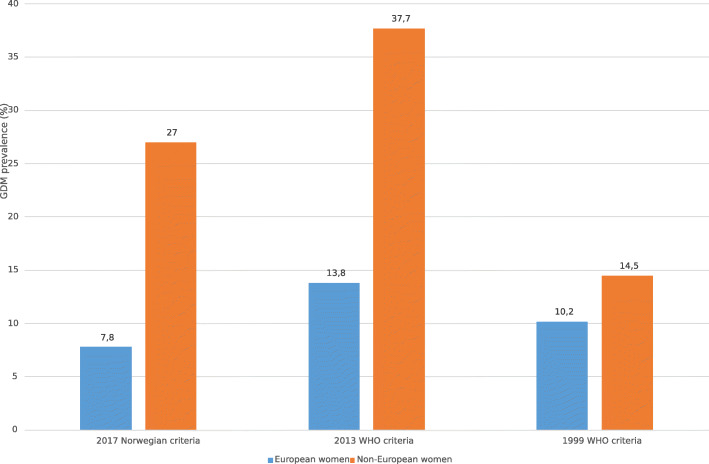
GDM prevalence based on three diagnostic criteria (_2017_Norwegian, _2013_WHO, _1999_WHO) for European and non-European women

The prevalence rates in European women compared to non-European women were 10.2 vs 14.5%, 13.8 vs 37.7% and 7.8 vs 27.0%, applying the _1999_WHO, _2013_WHO and _2017_Norwegian criteria, respectively (Fig. 2).

Compared with the non-GDM group, women diagnosed with GDM by either criteria were more likely to be older, heavier, shorter and of non-European origin (Supporting information Table S1, additional file 1). Moreover, using the _2017_Norwegian criteria, while 25.5% of women without GDM had overweight or obesity (BMI > 25 kg/m^2^), this was observed in 51.3% of women with GDM (*P* < 0.001). There were more primiparas in the non-GDM group (*P <* 0.001), except when applying the _1999_WHO criteria.

In logistic regression analyses, all selected variables except smoking, were significantly associated with GDM with the _2017_Norwegian criteria prior to adjustments (Table 3). Nevertheless, the associations observed for parity, education and height were strongly attenuated and lost their significance in the multivariable adjusted model 1. Age, pre-pregnancy BMI and ethnicity remained the only significant predictors in the final multivariable model (model 2). However, compared with women ≤25 years, an increased OR for developing GDM was only found for those above 35 years of age (aOR 1.73; 95% CI: 1.07–2.80; *P* < 0.026).

**Table 3:** Associations between maternal risk factors and gestational diabetes mellitus in univariate analysis and multivariate analysis, applying the _2017_Norwegian criteria

Variables	Univariate analysis			Model 1ª: r^2^ = 0.158			Model 2^є^r^2^ = 0.157		
	Odds ratio	95% CI	*P*-value	aOR	95% CI	*P*-value	aOR	95% CI	*P*-value
Age (years)			0.001			0.003			0.003
≤ 25	1			1			1		
25–29.9	0.76	0.52–1.14	0.191	0.90	0.57–1.41	0.651	0.86	1.20–2.58	0.495
30–34.9	0.82	0.55–0.21	0.319	1.07	0.67–1.71	0.768	1.00	0.64–1.56	0.984
≥ 35	1.42	0.93–2.16	0.100	1.84	1.10–3.07	0.020	1.73	1.07–2.80	0.026
Pre-pregnancy BMI (kg/m^2^)			< 0.001			< 0.001			< 0.001
≤ 25	1			1			1		
25–26.9	1.86	1.30–2.66	0.001	1.76	1.20–2.57	0.004	1.77	1.21–2.58	0.003
27–29.9	2.73	1.89–3.95	< 0.001	2.60	1.75–3.87	< 0.001	2.64	1.78–3.92	< 0.001
≥ 30	5.97	4.31–8.26	< 0.001	4.96	3.43–7.16	< 0.001	5.12	3.56–7.35	< 0.001
non-European ethnicity	4.36	3.35–5.69	< 0.001	2.46	1.53–3.94	< 0.001	2.72	1.78–4.13	< 0.001
Parous	1.53	1.21–1.94	< 0.001	0.99	0.73–1.34	0.955			
Education						0.725			
Higher education	1			1					
high school education	2.07	1.59–2.71	< 0.001	1.12	0.79–1.58	0.504			
primary or less	4.60	3.11–6.80	< 0.001	1.21	0.69–2.09	0.509			
Height (cm)	0.95	0.93–0.96	< 0.001	0.99	0.96–1.01	0.341			
Current smoker	1.66	0.89–3.11	0.112	1.29	0.65–2.56	0.454			
Cohort									
Stork Rikshospitalet	1								
FFF	0.77	0.52–1.15	0.200						
STORK Groruddalen	2.70	2.03–3.57	< 0.001						
TRIP	0.41	0.26–0.64	< 0.001						
Diabetes in family*	1.88	1.40–2.53	< 0.001						

Binary logistic regression was performed, _2017_Norway criteria

^a^*Adjusted for age, pre-pregnancy BMI, ethnicity, parity, education, height, smoking and cohort (not shown)*

^*є*^*Adjusted for age, pre-pregnancy BMI, ethnicity and cohort (not shown)*

Abbrevations: *aOR* adjusted odds ratio, *CI* confidens interval, *BMI* body mass index

* Not measured in Fit for Delivery

Applying the _2013_WHO criteria led to similar findings (Table 4). For the _1999_WHO, however, non-European ethnicity was not significantly associated with GDM, while parity and height remained significant in the final adjusted model (Supporting information Table S2, additional file 1). The predictive power of all models was low, with Nagelkerke values ranging from 0.9 to 16.4%, depending on the criteria applied. Sensitivity analysis restricted to individuals without lifestyle intervention in two of the cohorts led to similar findings, although age was no longer significant (not shown).

**Table 4:** Associations between maternal risk factors and gestational diabetes mellitus in univariate analysis and multivariate analysis, applying the _2013_WHO criteria

Variables	Univariate analysis			Model 1ª: r^2^ = 0.164			Model 2^є^r^2^ = 0.163		
	Odds ratio	95% CI	*P*-value	aOR	95% CI	*P*-value	aOR	95% CI	*P*-value
Age (years)			< 0.001			< 0.001			< 0.001
≤ 25	1			1			1		
25–29.9	0.74	0.53–1.01	0.062	0.91	0.63–1.32	0.622	0.87	0.60–1.25	0.445
30–34.9	0.85	0.62–1.17	0.321	1.24	0.84–1.83	0.267	1.18	0.82–1.70	0.381
≥ 35	1.50	1.07–2.13	0.020	2.17	1.42–3.33	< 0.001	2.07	1.38–3.09	< 0.001
Pre-pregnancy BMI (kg/m^2^)			< 0.001			< 0.001			< 0.001
≤ 25	1			1			1		
25–26.9	1.84	1.38–2.46	< 0.001	1.69	1.24–2.30	0.001	1.70	1.25–2.32	< 0.001
27–29.9	3.23	2.40–4.35	< 0.001	2.96	2.15–4.09	< 0.001	3.02	2.18–4.16	0.001
≥ 30	4.84	3.60–6.49	< 0.001	3.90	2.80–5.43	< 0.001	4.06	2.93–5.61	< 0.001
non-European ethnicity	3.79	3.00–4.78	< 0.001	1.91	1.28–2.86	0.002	2.17	1.51–3.10	< 0.001
Parous	1.49	1.23–1.80	< 0.001	1.02	0.79–1.31	0.875			
Education						0.340			
Higher education	1			1					
high school education	1.89	1.52–2.36	< 0.001	1.43	0.88–2.33	0.148			
primary or less	4.17	2.94–5.92	< 0.001	1.11	0.84–1.47	0.474			
Height	0.96	0.94–0.97	< 0.001	0.99	0.98–1.00	0.580			
Current smoker	0.65	0.38–1.12	0.119	1.13	0.98–1.01	0.674			
Cohort			< 0.001						
Stork Rikshospitalet	1								
FFF	0.94	0.69–1.26	0.668						
STORK G	2.64	2.09–3.34	< 0.001						
TRIP	0.41	0.29–0.59	< 0.001						
Diabetes in family *	1.85	1.44–2.38	< 0.001						

Binary logistic regression was performed, _2013_WHO-criteria. ^a^*Adjusted for age, pre-pregnancy BMI, ethnicity, parity, education, height, smoking and cohort (not shown).*^*є*^*Adjusted for age, pre-pregnancy BMI, ethnicity and cohort (not shown)*

Abbrevations: *aOR* adjusted odds ratio, *CI* confidens interval, *BMI* body mass index

* Not measured in Fit for Delivery

Table 5 displays estimates of sensitivity and the proportion needed to be screened for selected risk factors combinations, stratified for ethnic origin. In European women, the combination “age≥25 years or BMI≥25 kg/m^2^” achieved the highest sensitivity of 96.5% (i.e. detected 96.5% of GDM cases), but because these risk factors occurred in 93%, an OGTT would be required in almost all women. By adding parity to the age thresholds (25 years for primipara and 35 years for parous) the number of OGTT needed was reduced to 75%, although a reduction in sensitivity to 85% was observed. Similar trends were observed for women with non-European background, except that family history of diabetes achieved a higher sensitivity (42.6%) than in their European counterparts (11%). Overall, the sensitivity of the risk factors was slightly higher when applying the _2017_Norwegian criteria than the _2013_WHO.

**Table 5:** Performance of risk factors, alone or in combination, for the identification of GDM, with two criteria (2017 Norwegian and 2013 WHO)

***2017 Norwegian criteria***						
**Risk factors**	**Sensitivity (%)**	**Specificity (%)**	**PPV (%)**	**NPV (%)**	**OGTT’s needed (%)**	**Undetected GDM cases (%)**
**European background,*n*** **= 2588**						
BMI ≥ 25 kg/m^2^	53.0	75.6	15.5	95	26.6	47.0
BMI ≥ 27 kg/m^2^	36.6	87	19.2	94.2	14.9	63.4
BMI ≥ 30 kg/m^2^	22.8	94.4	24.4	93.9	6.8	77.7
Age ≥ 25 years	91.1	10.1	7.9	93.1	90.0	8.9
Age ≥ 30 years	60.4	45.5	8.6	93.1	54.9	39.6
Age ≥ 25 or BMI ≥ 25	96.5	7.2	8.1	96.1	93.1	3.5
Age ≥ 30 or BMI ≥ 30	71.3	42.7	9.5	94.6	58.4	28.7
Age ≥ 30 or BMI ≥ 27	74.3	39.4	9.4	94.8	61.6	25.7
BMI ≥ 25 or (Primipara + Age ≥ 25) or (parous + age ≥ 35)	85.1	26.0	8.9	95.4	74.9	14.9
BMI ≥ 25 or (Primipara + Age ≥ 25) or (parous + age ≥ 40)	78.7	32.3	9.0	94.7	68.5	21.3
BMI ≥ 25 or (Primipara + Age ≥ 30) or (parous + age ≥ 35)	72.8	47.9	10.6	95.4	53.7	27.2
BMI ≥ 27 or (Primipara + Age ≥ 30) or (parous + age ≥ 35)	61.9	54.5	10.3	94.4	46.8	38.1
Family history of diabetes*	11.5	89.1	8.7	92.1	10.6	87.3
BMI ≥ 25 or Age ≥ 30 or family history of diabetes	87.8	22.8	9.3	95.4	78.1	12.2
**Non-European background,*n*** **= 393**						
BMI ≥ 23 kg/m^2^	64.2	46.0	30.5	77.6	56.7	35.8
BMI ≥ 25 kg/m^2^	47.2	66.2	34.0	77.2	37.4	52.8
BMI ≥ 27 kg/m^2^	37.7	80.5	41.7	77.8	24.4	62.3
Age ≥ 25 years	80.2	23.3	27.9	76.1	77.6	19.8
Age ≥ 30 years	50.0	63.4	33.5	77.4	40.2	50.0
Age ≥ 25 or BMI ≥ 25	87.7	17.4	28.2	79.4	84.0	12.3
Age ≥ 30 or BMI ≥ 23	74.5	33.1	29.2	77.9	69.0	25.5
BMI ≥ 25 or (Primipara + Age ≥ 25) or (parous + age ≥ 35)	69.8	42.5	31.0	79.2	60.8	30.2
Family history of diabetes*	42.6	64.3	30.3	75.4	37.6	57.4
***2013 WHO criteria***						
**Risk factors**	**Sensitivity (%)**	**Specificity (%)**	**PPV (%)**	**NPV (%)**	**OGTT’s needed (%)**	**Undetected GDM cases (%)**
**European background,*n*** **= 2588**						
BMI ≥ 25 kg/m^2^	48.3	76.8	25.0	90.3	26.6	51.7
BMI ≥ 27 kg/m^2^	33.2	87.9	29.5	89.7	14.9	66.8
BMI ≥ 30 kg/m^2^	16.6	94.8	33.7	87.7	6.8	83.4
Age ≥ 25 years	90.4	10.1	13.8	86.9	90.0	9.6
Age ≥ 30 years	62.1	46.2	15.5	88.4	54.9	37.9
Age ≥ 25 or BMI ≥ 25	95.2	7.2	14.1	90.4	93.1	4.8
Age ≥ 30 or BMI ≥ 30	69.7	43.4	16.4	90.0	58.4	30.3
Age ≥ 30 or BMI ≥ 27	75.0	40.5	16.7	91.0	61.6	25.0
BMI ≥ 25 or (Primipara + Age ≥ 25) or (parous + age ≥ 35)	83.1	26.4	15.3	90.8	74.9	16.9
BMI ≥ 25 or (Primipara + Age ≥ 25) or (parous + age ≥ 40)	76.4	32.7	15.3	89.7	68.5	23.6
BMI ≥ 25 or (Primipara + Age ≥ 30) or (parous + age ≥ 35)	71.1	49.1	18.2	91.4	53.7	28.9
BMI ≥ 27 or (Primipara + Age ≥ 30) or (parous + age ≥ 35)	62.9	55.8	18.5	90.4	46.8	37.1
Family history of diabetes*	13.2	89.5	17.3	86.6	10.6	86.8
BMI ≥ 25 or Age ≥ 30 or family history of diabetes	87.5	23.5	16.2	91.8	78.1	12.5
**Non-European background,*n*** **= 393**						
BMI ≥ 23 kg/m^2^	66.2	49.0	43.9	70.6	56.7	33.8
BMI ≥ 25 kg/m^2^	47.3	68.6	47.6	68.3	37.4	52.7
BMI ≥ 27 kg/m^2^	36.5	89.2	56.3	68.4	24.4	63.5
Age ≥ 25 years	80.4	24.1	39.0	67.0	77.6	19.6
Age ≥ 30 years	48.0	64.5	44.9	67.2	40.2	52.0
Age ≥ 25 or BMI ≥ 25	87.8	18.4	39.4	71.4	84.0	12.2
Age ≥ 30 or BMI ≥ 23	76.4	35.5	41.7	71.3	69.0	23.6
BMI ≥ 25 or (Primipara + Age ≥ 25) or (parous + age ≥ 35)	69.6	44.5	43.1	70.8	60.8	30.4
Family history of diabetes*	40.1	64.0	40.1	64.0	37.6	59.9

Abbrevations: *BMI* body mass index, *WHO* World Health Organization, *PPV* positive predictive value, *NPV* negative predictive value, *OGTT* oral glucose tolerance test, *GDM* gestational diabetes

*Family history of diabetes was not measured in Fit for Delivery

**Fig. 3:**
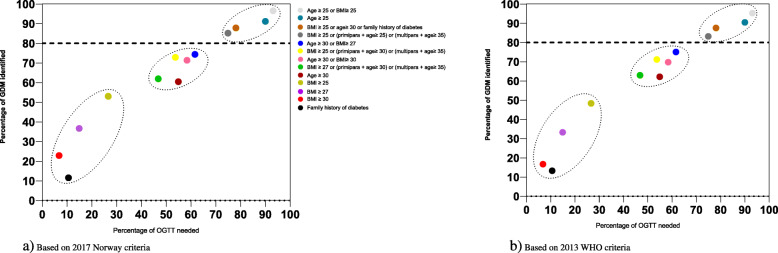
Screening performance (sensitivity and percentage offered an OGTT) of risk factors (single or in combinations) for European women with the _2017_Norwegian criteria (a) and _2013_WHO criteria (b). The color of the points indicates the risk factors used, and the line indicates 80% sensitivity. The clusters indicate poor (bottom left corner), moderate and good performance (top right corner)

Figure 3 shows the proportion of correctly identified GDM cases for European women, and proportion that would be offered an OGTT for each risk factor or combination of factors by the _2017_Norwegian and _2013_WHO criteria. Irrespective of the risk factor used, the sensitivity increased with the number of women needing a test for both diagnostic criteria, displaying three clusters of four to five factors with poor, moderate and good performance. To identify at least 80% of women with GDM (good performance), at least 75% of all women would need to undergo an OGTT. The risk factor displaying both high sensitivity and the smallest proportion of OGTT’s, was the combination “BMI≥25 kg/m^2^ or (primipara+age≥25) or (parous+age≥35)”. With 75% requiring a test, this factor combination failed to identify 15% of women with GDM by _2017_Norwegian criteria. The proportion of OGTT required could be reduced to 54% by increasing the threshold for age to ≥30 years for primipara (moderate performance); however, this approach implies that 27% of women with GDM will remain undiagnosed (Table 5).

## Discussion

In this study of women universally offered an OGTT during the second half of pregnancy, we found a similar overall prevalence of GDM (10.7% vs 10.3%) with the _2017_Norwegian criteria and the previously used criteria (_1999_WHO), but using lower glucose level thresholds in line with _2013_WHO criteria, identified considerably higher numbers of women with GDM (16.9%). The prevalence more than doubled for non-European women applying the _2013_WHO and _2017_Norwegian criteria, even after adjusting for covariates. Our study further shows that while advancing age and elevated BMI increased the risk of GDM, using these risk factors in pre-diagnostic screening is a poor method for accurately identifying women with GDM, resulting in many missed cases unless 70–80% of European women are tested. The sensitivity of the risk factors was lower for non-European women, indicating an even stronger rationale for universal screening in these women.

Although shifting from the older _1999_WHO criteria to the new _2017_Norwegian criteria resulted in a similar frequency of GDM, the groups identified differ in terms of their metabolic profile. The latter criteria identified more women with a higher pre-pregnancy BMI and non-European ethnicity, presumably attributable to the lower fasting glucose threshold.

Our prevalence rates applying the _2013_WHO criteria are comparable with estimates reported in other studies in the past decade, although differences in screening procedures, demographic characteristics of the subjects as well as the ethnic make-up of the population make direct comparisons complex. Guariguata et al. [15] estimated that the global prevalence of hyperglycaemia using the _2013_WHO criteria was 16.9%. A more recent meta-analysis of high-income countries in Europe found an overall GDM prevalence of 5.4%, regardless of diagnostic criteria used [16]. In contrast, a study using _2013_WHO thresholds and only fasting glucose in a Danish pregnancy cohort, found that 40% were classified as having GDM [17]. The authors raised important questions about uniform application of diagnostic thresholds across the world, and suggested population-based local recommendations.

Multiple studies have evaluated selective risk factor-based strategies aiming to identify the best diagnostic approach for GDM [18, 19]. We demonstrate that the most sensitive and specific cut-offs for maternal age and BMI in European women were age ≥ 25 years and BMI ≥25 kg/m^2^ when parity was added. However, used as a screening strategy this would mean inviting the majority of women for an OGTT as at least one of these risk factors applies to most women today. This confirms recent findings from a systematic review and meta-analysis by Farrar et al. [8] concluding that sensitivity increases with the number of women needing a test. This strategy does not vary much from universal screening, and supports the contention that identification of GDM requires testing of almost all pregnant women [20] especially considering the rise in maternal age and overweight/obesity among childbearing over recent years [21].

Selective screening has the potential to spare many pregnant women of diagnostic testing thereby reducing time and resource use. However, consistent with others [22, 23] we found that screening on the basis of risk factors would result in a larger number of missed diagnoses and hence limit the opportunity for immediate and long-term follow up and treatment. This is of concern, as a substantial proportion of women with GDM have no defined risk factors [24, 25]. The importance of GDM management is now widely accepted, and evidence supports that treatment of even milder degrees of hyperglycaemia could improve pregnancy outcomes [26, 27]. Additionally, universal screening has the unique potential to identify this subset of women who would not otherwise be identified as having GDM, and, therefore, provide clinicians, as well as the women themselves, an opportunity to plan postpartum lifestyle interventions that could prevent or delay the onset of future type 2 diabetes [28–30].

Our study has several strengths. We merged data from four contemporary birth cohorts, allowing more powerful and flexible analyses. Additionally, although the level of missing was generally low, missing data were adequately handled by multiple imputation to prevent biased results. By including different geographical populations in Norway, we believe that the results may be broadly generalizable in Norway as well as to different antenatal populations in other high-income countries. Moreover, our study included women from various ethnic groups, making our findings relevant to other European countries with similar immigrant populations. It is of note, however, that almost all non-European women came from one study and more than half were of Asian (mainly South Asian) origin. Nevertheless, the proportion included and the composition of this group, is representative for the pregnant population with non-European ethnicity living in Norway [31].

The majority of the European women in our study had a normal BMI and high educational level, which may indicate that our prevalence rates of GDM are less generalizable to more high-risk populations. The rates of overweight and obesity in our cohort were somewhat lower than our background population (8% obesity in our study vs 12% nationally in 2018) [32]. A selection bias towards inclusion of individuals with a higher health awareness, as is often seen in clinical studies, may have led to underestimation of the reported prevalence rates and the numbers needed to be screened. A higher proportion of overweight/obesity would require an OGTT of a larger number of women. In addition, had a 1-h value been measured in our study, the prevalence of GDM by the _2013_WHO criteria would presumably have increased somewhat. Second, two of the included studies were RCT’s with a lifestyle intervention for half of the women. However, no effect of the intervention on GDM status was reported in these studies and, reassuringly, our findings remained unchanged in sensitivity analyses. Lastly, we present data from four cohorts pooled into one data set where each study differs somewhat in terms of inclusion period, time of OGTT and geography, although by including Norwegian studies only and adjusting for study cohort this source of heterogeneity was limited.

## Conclusion

The use of a stricter diagnostic criteria than the _2013_WHO (OR of 2.0 vs. 1.75) limited the prevalence of GDM to approximately the same level as the older _1999_WHO. We found that maternal characteristics are of limited use in identifying women with GDM, requiring testing of almost all women to avoid overlooking a substantial number of cases. The costs and benefits of universal screening, and the use of alternative testing algorithms or biomarkers, require further evaluation.

## Supplementary Information

**Additional file 1.** : Supporting information Table S1. Characteristics of study participants according to their glucose tolerance status, with three criteria (_1999_WHO, _2013_WHO and _2017_Norwegian criteria). Supporting information Table S2. Associations between maternal risk factors and gestational diabetes mellitus in univariate analysis and multivariate analysis, using the _1999_WHO criteria.

## Data Availability

The datasets generated and/or analyzed during the current study are not publicly available due to the dataset containing clinical data which cannot be shared publicly, and as the study is part of a PhD work. The data are available from the corresponding author on reasonable request.

## Abbreviations

BMI: Body mass index
GDM: Gestational diabetes mellitus
OGTT: Oral glucose tolerance test
OR: Odds ratio
WHO: World Health Organization

## References

[1] American Diabetes Association (2018). 2. Classification and diagnosis of diabetes: standards of medical Care in Diabetes—2018. Diabetes Care.

[2] Song C, Lyu Y, Li C, Liu P, Li J, Ma RC (2018). Long-term risk of diabetes in women at varying durations after gestational diabetes: a systematic review and meta-analysis with more than 2 million women. Obes Rev.

[3] Crowther CA, Hiller JE, Moss JR, McPhee AJ, Jeffries WS, Robinson JS (2005). Effect of treatment of gestational diabetes mellitus on pregnancy outcomes. New Engl J Med.

[4] Benhalima K, Mathieu C, Assche A, Damm P, Devlieger R, Mahmood T (2016). Survey by the European board and College of Obstetrics and Gynaecology on screening for gestational diabetes in Europe. Eur J Obstet Gynecol Reprod Biol.

[5] Metzger BE, Lowe LP, Dyer AR, Trimble ER, Chaovarindr U, Coustan DR (2008). Hyperglycemia and adverse pregnancy outcomes. N Engl J Med.

[6] Cundy T, Ackermann E, Ryan EA (2014). Gestational diabetes: new criteria may triple the prevalence but effect on outcomes is unclear. BMJ..

[7] Marozio L, Picardo E, Filippini C, Mainolfi E, Berchialla P, Cavallo F, et al. Maternal age over 40 years and pregnancy outcome: a hospital-based survey. J Matern Fetal Neonatal Med. 2019;32(10):1602–8. 10.1080/14767058.2017.1410793. 10.1080/14767058.2017.1410793 29216770

[8] Farrar D, Simmonds M, Bryant M, Lawlor DA, Dunne F, Tuffnell D, Sheldon TA (2017). Risk factor screening to identify women requiring oral glucose tolerance testing to diagnose gestational diabetes: a systematic review and meta-analysis and analysis of two pregnancy cohorts. PLoS One.

[9] Jenum AK, Sletner L, Voldner N, Vangen S, Mørkrid K, Andersen LF, Nakstad B, Skrivarhaug T, Rognerud-Jensen OH, Roald B, Birkeland KI (2010). The STORK Groruddalen research programme: a population-based cohort study of gestational diabetes, physical activity, and obesity in pregnancy in a multiethnic population. Rationale, methods, study population, and participation rates. Scand J Public Health.

[10] Frøslie KF, Røislien J, Qvigstad E, Godang K, Bollerslev J, Voldner N (2013). Shape information from glucose curves: functional data analysis compared with traditional summary measures. BMC Med Res Methodol.

[11] Sagedal LR, Øverby NC, Lohne-Seiler H, Bere E, Torstveit MK, Henriksen T, Vistad I (2013). Study protocol: fit for delivery - can a lifestyle intervention in pregnancy result in measurable health benefits for mothers and newborns? A randomized controlled trial. BMC Public Health.

[12] Stafne SN, Salvesen KÅ, Romundstad PR, Eggebø TM, Carlsen SM, Mørkved S (2012). Regular exercise during pregnancy to prevent gestational diabetes: a randomized controlled trial. Obstet Gynecol.

[13] Van Buuren S (2018). Flexible imputation of missing data.

[14] Steyerberg EW (2009). Clinical prediction models.

[15] Guariguata L, Linnenkamp U, Beagley J, Whiting DR, Cho NH (2014). Global estimates of the prevalence of hyperglycaemia in pregnancy. Diabetes Res Clin Pract.

[16] Eades CE, Cameron DM, Evans JMM (2017). Prevalence of gestational diabetes mellitus in Europe: a meta-analysis. Diabetes Res Clin Pract.

[17] McIntyre HD, Jensen DM, Jensen RC, Kyhl HB, Jensen TK, Glintborg D (2018). Gestational diabetes mellitus: does one size fit all? A challenge to uniform worldwide diagnostic thresholds. Diabetes Care.

[18] Pintaudi B, Di Vieste G, Corrado F, Lucisano G, Pellegrini F, Giunta L (2014). Improvement of selective screening strategy for gestational diabetes through a more accurate definition of high-risk groups. Eur J Endocrinol.

[19] Benhalima K, Damm P, Van Assche A, Mathieu C, Devlieger R, Mahmood T (2016). Screening for gestational diabetes in Europe: where do we stand and how to move forward?: a scientific paper commissioned by the European board &amp; College of Obstetrics and Gynaecology (EBCOG). Eur J Obstet Gynecol Reprod Biol.

[20] Hod M, Kapur A, Sacks DA, Hadar E, Agarwal M, Di Renzo GC (2015). The International Federation of Gynecology and Obstetrics (FIGO) initiative on gestational diabetes mellitus: a pragmatic guide for diagnosis, management, and care. Int J Gynaecol Obstet.

[21] Matthews TJ, Hamilton BE (2014). First births to older women continue to rise. NCHS Data Brief.

[22] Zhou Q, Wang Q, Shen H, Zhang Y, Zhang S, Li X (2017). Prevalence of diabetes and regional differences in Chinese women planning pregnancy: a Nationwide population-based cross-sectional study. Diabetes Care.

[23] Cosson E, Cussac-Pillegand C, Benbara A, Pharisien I, Jaber Y, Banu I, Nguyen MT, Valensi P, Carbillon L (2014). The diagnostic and prognostic performance of a selective screening strategy for gestational diabetes mellitus according to ethnicity in Europe. J Clin Endocrinol Metab.

[24] Avalos GE, Owens LA, Dunne F, Collaborators AD (2013). Applying current screening tools for gestational diabetes mellitus to a European population: is it time for change?. Diabetes Care.

[25] Arora D, Arora R, Sangthong S, Leelaporn W, Sangratanathongchai J (2013). Universal screening of gestational diabetes mellitus: prevalence and diagnostic value of clinical risk factors. J Med Assoc Thail.

[26] Landon MB, Spong CY, Thom E, Carpenter MW, Ramin SM, Casey B, Wapner RJ, Varner MW, Rouse DJ, Thorp JM, Sciscione A, Catalano P, Harper M, Saade G, Lain KY, Sorokin Y, Peaceman AM, Tolosa JE, Anderson GB (2010). A multicenter, randomized trial of treatment for mild gestational diabetes. Obstet Anesth Dig.

[27] Hartling L, Dryden DM, Guthrie A, Muise M, Vandermeer B, Donovan L (2013). Benefits and harms of treating gestational diabetes mellitus: a systematic review and meta-analysis for the U.S. preventive services task force and the National Institutes of Health Office of medical applications of research. Ann Intern Med.

[28] Xin Y, Davies A, McCombie L, Briggs A, Messow CM, Grieve E, Leslie WS, Taylor R, Lean MEJ (2019). Within-trial cost and 1-year cost-effectiveness of the DiRECT/counterweight-plus weight-management programme to achieve remission of type 2 diabetes. Lancet Diabetes Endocrinol.

[29] Aroda VR, Christophi CA, Edelstein SL, Zhang P, Herman WH, Barrett-Connor E, Delahanty LM, Montez MG, Ackermann RT, Zhuo X, Knowler WC, Ratner RE, for the Diabetes Prevention Program Research Group (2015). The effect of lifestyle intervention and metformin on preventing or delaying diabetes among women with and without gestational diabetes: the diabetes prevention program outcomes study 10-year follow-up. J Clin Endocrinol Metab.

[30] Bao W, Tobias DK, Bowers K, Chavarro J, Vaag A, Grunnet LG, Strøm M, Mills J, Liu A, Kiely M, Zhang C (2014). Physical activity and sedentary behaviors associated with risk of progression from gestational diabetes mellitus to type 2 diabetes mellitus: a prospective cohort study. JAMA Intern Med.

[31] Norwegian Institute of Public Health The Medical Birth Registry of Norway - statistics. F18a: Mors fødeland, 2018, [Mothers country of birth]. Available online at: http://statistikkbank.fhi.no/mfr/ (Accessed 10th May 2021).

[32] Norwegian Institute of Public Health The Medical Birth Registry of Norway - statistics. F18a: Mors kroppsmasseindeks før svangerskapet, 2018 [Mothers body mass index pre pregnancy]. Available online at: http://statistikkbank.fhi.no/mfr/ (Accessed 3 Jan 2021).

